# Supplementary material for: Genetic Characterisation and Comparison of Three Human Coronaviruses (HKU1, OC43, 229E) from Patients and Bovine Coronavirus (BCoV) from Cattle with Respiratory Disease in Slovenia
Source: Viruses. 2021 Apr 15;13(4):676. doi: 10.3390/v13040676 (PMC8071153; doi:10.3390/v13040676)
Supplement: Supplementary file 1 [file viruses-13-00676-s001.zip › viruses-1176565-supplementary.pdf]

Genetic characterisation and comparison of three human coronaviruses (HKU1, OC43, 229E) from patients and bovine coronavirus (BCoV) from cattle with respiratory disease in Slovenia

Table S1. GenBank accession numbers associated with the sequences determined in this study.

No of sequence	Date of sample collection	Host	Clinical observation	Name of sample	Accession Number
Seq1	December 22, 2012	cattle	pneumonia	BCoV/SLO/TP14/2012	KX059608
Seq2	January 21, 2014	cattle	pneumonia	BCoV/SLO/TP66/2014	KX059609
Seq3	February 12, 2014	cattle	pneumonia	BCoV/SLO/TP73/2014	KX059610
Seq4	May 21, 2013	cattle	respiratory	BCoV/SLO/TZ95/2013	KX059611
Seq5	May 21, 2013	cattle	respiratory	BCoV/SLO/TZ98/2013	KX059612
Seq6	May 21, 2013	cattle	respiratory	BCoV/SLO/TZ96/2013	KX059613
Seq7	May 21, 2013	cattle	respiratory	BCoV/SLO/TZ94/2013	KX059614
Seq8	November 22, 2012	cattle	respiratory	BCoV/SLO/TZ15/2012	KX059615
Seq9	May 21, 2013	cattle	respiratory	BCoV/SLO/TZ102/2013	KX059616
Seq10	May 21, 2013	cattle	respiratory	BCoV/SLO/TZ101/2013	KX059617
Seq11	May 21, 2013	cattle	pneumonia	BCoV/SLO/TP49/2013	KX059618
Seq12	February 18, 2013	cattle	pneumonia	BCoV/SLO/TP36/2013	KX059619
Seq13	July 16, 2013	cattle	diarrhoea	BCoV/SLO/6906/2013	KX059620
Seq14	May 21, 2013	cattle	diarrhoea	BCoV/SLO/5580/2013	KX059621
Seq15	January 7, 2015	cattle	diarrhoea	BCoV/SLO/JJ-5/2015	KX059622
Seq16	January 21, 2015	cattle	diarrhoea	BCoV/SLO/JJ-6/2015	KX059623
Seq17	December 29, 2015	cattle	diarrhoea	BCoV/SLO/41376/2015	KX059624
Seq18	January 18, 2014	cattle	pneumonia	BCoV/SLO/TP63/2014	KX059625
Seq19	December 28, 2013	cattle	respiratory	BCoV/SLO/TZ103/2013	KX059626
Seq20	December 28, 2013	cattle	respiratory	BCoV/SLO/TZ104/2013	KX059627
Seq21	April 17, 2014	cattle	respiratory	BCoV/SLO/TZ132/2014	KX059628
Seq22	February 1, 2013	cattle	pneumonia	BCoV/SLO/TP30/2013	KX059629
Seq23	January 23, 2014	cattle	respiratory	BCoV/SLO/TZ65/2014	KX059630
Seq24	January 23, 2014	cattle	respiratory	BCoV/SLO/TZ66/2014	KX059631
Seq25	December 24, 2015	human	respiratory	OC43/SLO/60954/2015	KX059632
Seq26	February 4, 2016	human	respiratory	OC43/SLO/62706/2016	KX059633
Seq27	December 9, 2013	human	respiratory	OC43/SLO/39776/2013	KX059634
Seq28	December 13, 2013	human	respiratory	OC43/SLO/39849/2013	KX059635
Seq29	February 3, 2014	human	respiratory	OC43/SLO/40296/2014	KX059636
Seq30	January 18, 2014	human	respiratory	OC43/SLO/40760/2014	KX059637
Seq31	March 27, 2014	human	respiratory	OC43/SLO/43970/2014	KX059638
Seq32	March 26, 2014	human	respiratory	OC43/SLO/43979/2014	KX059639
Seq33	April 8, 2014	human	respiratory	OC43/SLO/44364/2014	KX059640
Seq34	August 14, 2014	human	respiratory	OC43/SLO/46580/2014	KX059641
Seq35	November 16, 2015	human	respiratory	OC43/SLO/59988/2015	KX059642
Seq36	November 19, 2015	human	respiratory	OC43/SLO/60116/2015	KX059643
Seq37	December 2, 2015	human	respiratory	OC43/SLO/60430/2015	KX059644
Seq38	December 28, 2015	human	respiratory	OC43/SLO/61010/2015	KX059645
Seq39	January 7, 2016	human	respiratory	OC43/SLO/61412/2016	KX059646
Seq40	January 8, 2016	human	respiratory	OC43/SLO/61480/2016	KX059647
Seq41	January 11, 2016	human	respiratory	OC43/SLO/61531/2016	KX059648
Seq42	January 20, 2016	human	respiratory	OC43/SLO/62066/2016	KX059649
Seq43	February 5, 2016	human	respiratory	OC43/SLO/62794/2016	KX059650
Seq44	February 20, 2016	human	respiratory	OC43/SLO/63863/2016	KX059651
Seq45	January 8, 2016	human	respiratory	OC43/SLO/61445/2016	KX059652

No of sequence	Date of sample collection	Host	Clinical observation	Name of sample	Accession Number
Seq46	January 31, 2016	human	respiratory	OC43/SLO/62519/2016	KX059653
Seq47	January 27, 2014	human	respiratory	OC43/SLO/41165/2014	KX059654
Seq48	January 29, 2014	human	respiratory	OC43/SLO/41313/2014	KX059655
Seq49	February 3, 2014	human	respiratory	OC43/SLO/41459/2014	KX059656
Seq50	19.February 19, 2014	human	respiratory	OC43/SLO/42347/2014	KX059657
Seq51	March 12, 2014	human	respiratory	OC43/SLO/43431/2014	KX059658
Seq52	April 22, 2014	human	respiratory	OC43/SLO/44656/2014	KX059659
Seq53	May 17, 2014	human	respiratory	OC43/SLO/45151/2014	KX059660
Seq54	May 232014	human	respiratory	OC43/SLO/45283/2014	KX059661
Seq55	February 21, 2010	human	respiratory	OC43/SLO/14041/2010	KX059662
Seq56	December 17, 2015	human	respiratory	HKU1/SLO/60770/2015	KX059663
Seq57	January 25, 2016	human	respiratory	HKU1/SLO/62213/2016	KX059664
Seq58	February 28, 2016	human	respiratory	HKU1/SLO/63860/2016	KX059665
Seq59	December 31, 2015	human	respiratory	HKU1/SLO/61443/2015	KX059666
Seq60	April 4, 2010	human	respiratory	HKU1/SLO/20580/2010	KX059667
Seq61	January 3, 2014	human	respiratory	HKU1/SLO/40304/2014	KX059668
Seq62	February 14, 2014	human	respiratory	HKU1/SLO/42056/2014	KX059669
Seq63	February 18, 2014	human	respiratory	HKU1/SLO/42284/2014	KX059670
Seq64	March 12, 2014	human	respiratory	HKU1/SLO/43609/2014	KX059671
Seq65	November 17, 2015	human	respiratory	HKU1/SLO/60004/2015	KX059672
Seq66	December 2, 2015	human	respiratory	HKU1/SLO/60422/2015	KX059673
Seq67	December 3, 2015	human	respiratory	HKU1/SLO/60454/2015	KX059674
Seq68	December 7, 2015	human	respiratory	HKU1/SLO/60558/2015	KX059675
Seq69	December 11, 2015	human	respiratory	HKU1/SLO/60641/2015	KX059676
Seq70	December 13, 2015	human	respiratory	HKU1/SLO/60668/2015	KX059677
Seq71	December 17, 2015	human	respiratory	HKU1/SLO/60781/2015	KX059678
Seq72	December 21, 2015	human	respiratory	HKU1/SLO/60867/2015	KX059679
Seq73	December 29, 2015	human	respiratory	HKU1/SLO/61107/2015	KX059680
Seq74	January 8, 2016	human	respiratory	HKU1/SLO/61456/2016	KX059681
Seq75	January 15, 2016	human	respiratory	HKU1/SLO/61833/2016	KX059682
Seq76	Janaury 23, 2016	human	respiratory	HKU1/SLO/62150/2016	KX059683
Seq77	January 26, 2016	human	respiratory	HKU1/SLO/62234/2016	KX059684
Seq78	Janaury 26, 2016	human	respiratory	HKU1/SLO/62261/2016	KX059685
Seq79	February 1, 2016	human	respiratory	HKU1/SLO/62556/2016	KX059686
Seq80	February 2, 2016	human	respiratory	HKU1/SLO/62637/2016	KX059687
Seq81	February 20, 2016	human	respiratory	HKU1/SLO/63673/2016	KX059688
Seq82	January 23, 2016	human	respiratory	HKU1/SLO/62197/2016	KX059689
Seq83	February 20, 2016	human	respiratory	HKU1/SLO/63615/2016	KX059690
Seq84	February 16, 2014	human	respiratory	HKU1/SLO/42161/2014	KX059691
Seq85	February 10, 2016	human	respiratory	HKU1/SLO/62985/2016	KX059692
Seq86	December 20, 2013	human	respiratory	HKU1/SLO/39995/2013	KX059693
Seq87	December 20, 2013	human	respiratory	HKU1/SLO/39999/2013	KX059694
Seq88	December 20, 2013	human	respiratory	HKU1/SLO/40019/2013	KX059695
Seq89	December 31, 2013	human	respiratory	HKU1/SLO/40216/2013	KX059696
Seq90	April 6, 2010	human	respiratory	229E/SLO/15756/2010	KX059697
